# Death of Alexander Barry

**Published:** 1832-11

**Authors:** 


### OBITUARY.

### DEATH OF ALEXANDER BARRY, ESQ., F.R.S.

This gentleman died at his residence in Furnival's Inn, on the 8th ultimo, in consequence of the severe injuries which he sustained, about a month since, in the performance of some chemical experiments on the gases. Mr. B. had been for five years a lecturer on chemistry and natural philosophy in Guy's Hospital, to which office he was appointed on the resignation of his uncle, Mr. W. Allen. The nature of the experiment in which he was engaged at the time he met with the fatal accident has not been correctly ascertained; but it is supposed, from his own statements, that he was wishing to try the effect of a powerfully expansile force in gases with their well-known inflammability: for this purpose he had condensed, by enormous pressure, the carburetted hydrogen, the sulphuretted hydrogen, and the carbonic oxide, in an iron recipient of about three-eighths of an inch in thickness; but whether the ignition of this compound took place through accident or design is unknown. It most probably originated from accident, since the explosion was heard immediately after a candle had been carried into his room. The particulars of this melancholy event have been already sufficiently made known, and it is therefore unnecessary to revert to them.

Mr. Barry had been but recently elected a fellow of the Royal Society, which distinguished honour he obtained through the presentation of a paper, published in the Philosophical Transactions for 1831, relative to the Chemical Action of Atmospheric Electricity on Saline Solutions. The results of his experiments on this subject are original and interesting, supplying another link to the chain of evidence which has been brought forward since the time of FRANKLIN to demonstrate the identity of atmospheric with ordinary electricity.

Those who were well acquainted with the excellent moral character and natural amiability of temper of this young and aspiring chemist will long regret that, through his great ardour in the pursuit of discovery, he should have thus been prematurely swept from the world of the living.

